# Operational Performance and Load Flexibility Analysis of Japanese Zero Energy House

**DOI:** 10.3390/ijerph18136782

**Published:** 2021-06-24

**Authors:** Xiaoyi Zhang, Weijun Gao, Yanxue Li, Zixuan Wang, Yoshiaki Ushifusa, Yingjun Ruan

**Affiliations:** 1 Innovation Institute for Sustainable Maritime Architecture Research and Technology, Qingdao University of Technology, Fushun Road 11, Qingdao 266033, China; c1dbb002@eng.kitakyu-u.ac.jp (X.Z.); weijun@kitakyu-u.ac.jp (W.G.); wangzixuan110133@126.com (Z.W.); 2 Faculty of Environmental Engineering, The University of Kitakyushu, Kitakyushu 808-0135, Japan; 3 Faculty of Economics and Business Administration, The University of Kitakyushu, Kitakyushu 802-8577, Japan; yi.619@163.com; 4 Institute of Mechanical Engineering, Tongji University, Siping Road 1239, Shanghai 200092, China; ruanyj@tongji.edu.cn

**Keywords:** zero energy house, operational performance, virtual energy storage, load flexibility

## Abstract

ZEHs (Zero Energy House) featuring energy-efficient designs and on-site renewable integration are being widely developed. This study introduced Japanese ZEHs with well-insulated thermal envelopes and investigated their detailed operational performances through on-site measurements and simulation models. Measurement data show that ZEHs effectively damped the variation of indoor air temperature compared to conventional houses, presenting great ability to retain inside heat energy, and are expected to potentially deliver energy flexibility as a virtual thermal energy storage medium. We developed a simplified thermal resistance–capacitance model for a house heating system; response behaviors were simulated under various scenarios. Results compared the variations of indoor temperature profiles and revealed the dependence of load flexibility on the building’s overall heat loss performance. We observed that overall heat loss rate played a crucial role in building heat energy storage efficiency; a well-insulated house shortened the heat-up time with less energy input, and extended the delayed period of indoor temperature under intermittent heating supply; a high set-point operative temperature and a low ambient temperature led to lower virtual thermal energy storage efficiency. The preheating strategy was simulated as an effective load-shifting approach in consuming surplus PV generation; approximately 50% of consumed PV generation could be shifted to replace grid import electricity for room heating during the occupied period.

## 1. Introduction

Greenhouse gas emission mitigation involves energy conservation and energy efficiency improvement. The Japanese government is aiming (i) to deliver net zero greenhouse gas emissions, and (ii) to be climate neutral by 2050. The building sector, as a major energy consumers, is expected to play an important role in meeting carbon emission reduction targets [1]. Currently, the residential sector is responsible for approximate 15% of final social energy consumption. The Japanese Ministry of Economy, Trade, and Industry (METI), to encourage the development of low-energy buildings, launched a net zero energy target, for all new homes to be built to net zero by 2030. A large number of zero energy houses (ZEHs) feature on-site renewable energy resources and higher thermal insulation levels when compared to the current energy efficiency standards [1,2]. Integration of on-site renewable energy resources, and growing electrification of building energy consumption, play key roles in achieving ZEH in the operational stage. However, there are existing challenges in dealing with real-time balances between on-site energy consumption and variable renewable generation profiles. One effective solution would be to increase the flexibility of the energy system, in terms of increasing self-consumption of local renewable energy [3,4]. Traditional approaches (in regards to coordinating with the challenges) involve implementation of energy storage dispatch systems, such as hot water tanks and battery units [5,6]. However, this may cause an increase in capacity investment.

The buildings sector is a major contributor of social energy consumption (e.g., due to highly flexible space heating and cooling loads), accounting for a large proportion of energy usage. High-efficient energy design standards are highlighted as minimizing heating or cooling energy consumption in ZEHs at the design stage, and are favorable in damping indoor temperature fluctuations. This is expected to compensate for variabilities in decentralized generation and consumption through coordinated demand side management strategies [2,7]. Demand response is considered to play an important role in delivering aggregated load-based flexibility services in the buildings sector [8,9]. Meanwhile, it is worth noting that increasing electrification to the end user offers the opportunity to harness energy flexibility from different aspects [10,11]. There are different approaches to energy conservation and flexibility exploration; more efforts are being dedicated to energy consumption management. Building thermal load management, through hot water usage, indoor space heating, or cooling consumption, is increasingly recognized as an important flexibility resource. Meanwhile, wide implementation of “behind smart meters”, high energy-efficient design standards, and improved connectivity between individual buildings and energy networks [12,13], provides real-time load shift opportunities, as virtual energy storage dispatches, and forces energy flexibility strategies to evolve. Policy makers or energy managers also need to acquire a deep understanding of operational performances of existing energy efficiency buildings.

The present research draws on the findings of a study on advanced zero energy houses (ZEHs) located in Kitakyushu, Japan. In order to acquire a deep understanding of how they operate, and to what extent energy flexibility exists in well-insulated houses, we combined the “behind smart meter” data and simulations to investigate the energy efficiency and flexibility performances of ZEHs in various scenarios. A potential load shifting strategy to facilitate operational energy flexibility was also analyzed (i.e., by considering increasing the local self-consumption rate of photovoltaic (PV)-generated electricity).

## 2. Related Work

It is worth noting that the definition of ZEH may vary by country, region, and group [14]. The ZEH concept was originally proposed in the 1970s, when Esbensen and Korsgaard used solar energy to satisfy the heating demands of a residential house in Denmark [15]. Ever since, ZEHs have been constructed throughout the world and the number of completed ZEH projects is growing. The European Directive defines nearly-ZEHs as energy efficient buildings with very low-energy demands, which are able to cover energy consumption using local, renewable energy [16,17]. In Canada, net ZEH is defined as a home that only uses as much energy as it can produce from on-site renewable energy [14]. The Japanese ZEH concept primarily involves achieving net annual energy consumption of around zero, by saving as much energy as possible, while maintaining indoor thermal comfort. Detailed energy-efficient measures generally include high-performance thermal insulation, uptake of energy efficient technology, and renewable energy integration. On-site generation, such as PV and fuel cell, plays a key role in the achievement of annual net energy balance. It is worth noting that the ratio of on-site PV generation to cover building electricity consumption is limited [18,19]. Real-time mismatch between simultaneous PV generation and residential load profiles may pose a challenge to the reliability of district grid operations [2]. Ongoing research, in regards to the energy flexibility of buildings, was initially motivated by rising energy efficiency design standards and challenges from high-penetration renewable integration [20,21]. Annex introduced the energy-flexible building concept, defined as buildings with the ability to manage their energy demand and generation, in accordance with climate conditions, demand, and grid requirements [22,23]. A high level of building load flexibility is expected to promote energy conservation and reduce energy costs; thus, facilitate the integration of on-site variable renewable generation. With respect to the reduction of primary energy consumption, full exploration of demand-side flexibility was highlighted towards nearly- or net-zero energy buildings [24].

Energy cost-savings benefits are associated with coordinated load management in response to dynamic electricity prices or incentive strategies [25]. The popularization of domestic energy storage units, smart meters, and Internet of Things systems motivate users’ active interactions to improve energy flexibility. Previous studies show that different alternatives could provide energy flexible services in the building sector, which can be classified into two types: one is adjustable technologies, such as a heat pump, CHP system, or energy storage unit, which could bring cost-saving benefits to customers under optimal, automated control environments [2,7]. The other is incentive-based demand response programs, in terms of dynamic electricity tariff or incentive payments—customers could be encouraged to schedule or suppress energy usage within a timespan when there are potential economic benefits [26,27]. The elasticity of an energy load reflects customer reactions to potential cost-savings. Studies have investigated rule-based control performances of buildings equipped with distributed energy resources, home devices, and storage units, including batteries and hot water tanks. For example, previous research from Li et al. [5] presented an optimized grid-tied PV-battery system; an optimized schedule of the battery charging/discharging flow raises the local PV self-consumption ratio, and in turn smooths the grid load in aggregated form. Yu et al. [28] proposed the optimized control of the lighting system at an office building, to provide potential flexibility, which could bring more than 30% lighting power capacity reduction. Electrical vehicles that were used to optimize the power exchange between multiple buildings and the main grid maximized PV self-consumption and reduced grid interaction; cost reduction was up to 8.1% through reducing grid import [29]. Aggregated control on the domestic residential heat pump verified that it could offer power modulation, providing upward or downward service [30].

Building thermal load presents a larger inertia than electricity consumption [31]; short-term fluctuations in heating, ventilation, and air conditioning (HVAC) energy use has less effect on indoor air temperature and thermal comfort, especially for the building envelope processed with better thermal insulation [32]. A thermostatically-controlled load is expected to offer a balancing service by allowing decreased or increased energy usage (than a given reference load profile) without jeopardizing indoor comfort [7,33]. A number of studies had activated and utilized building thermal mass to gain energy flexibility and economic benefits [4,34,35]. For instance, Short, M. et al. [35] presented a unit reallocation scheme for a HVAC system based on a price relationship in building stock; results illustrated the effectiveness of the flexible solution in reducing energy consumption and cost. Indoor–outdoor temperature gradient is the driving force for heat transfer between the building envelope and outdoor environment; an equivalent resistance–capacitance (RC) thermal model is widely accepted as a good approach to capture dynamic thermal behaviors within buildings [33,36,37]. Hurwitz Z L et al. modeled a one-dimensional building thermodynamic process and evidenced the effectiveness of cost saving and carbon emission reduction when flexible buildings integrated into a multi-energy system by allowing more indoor temperature comfort range [38]. Insight into the indoor–outdoor dynamic energy transfer process and heat energy store capability of the building thermal mass was assessed by adjusting indoor set-point temperatures; approximately 50% of the domestic heating peak load is shiftable within a temperature comfort range [39]. The indoor temperature of the building was optimized by managing the charging/discharging energy of the building’s virtual energy storage system; the thermal load shift brings more than an 8.0% free capacity for distributed generators in the hybrid energy system [40]. Thermal properties of a building envelope, such as thermal mass or insulation level, are generally considered to have a significant influence on the heat energy conservation and flexibility [41]. A building with low thermal insulation would lead to a low charge–discharge cycling efficiency, especially on cold days [42]. Reference [43] found that great envelope thermal inertial plays an important role in potentiating energy flexibility, especially under a high thermal load. Meanwhile, a building envelope with a better thermal insulation level is expected to provide a damping effect on air temperature changes and enable the building with a greater ability to retain indoor heat, resulting in load shifting potential for a long-lasting period [6,44].

In regards to real-time load balance regulation, energy-savings, and penetration maximization of variable renewable energy—there were some studies already conducted on energy flexibility [45]. Ramp rate and duration metrics have been applied to define the operational flexibility of large-scale power systems, and the flexibility properties of different power unit types have been quantified and compared [46]. Energy flexibility of buildings can be evaluated from different perspectives. Reference [47] revealed the load shifting efficiency and recommended the cost index to reveal the cost-effectiveness performance of the building’s energy flexibility. Operational flexibility from distributed energy resources generally comes at a positive cost through coordinated management [48]. The relationship between operational costs with an indoor thermal comfort band is graphically presented in the cost curve; the wide thermal comfort range indicates great cost-saving potential [49]. In [3], the authors described the temporal flexibility of a building energy system, in terms of time duration and energy capacity. Reference [50] proposed the energy flexibility characterization methodology to label the flexible index of a building’s energy system, evaluating how buildings could respond to flexibility requirements of a given power system.

Thermal insulation level, ambient condition, as well as local energy supply–demand balance would influence building energy consumption and load shift potential. In regards to the better energy-efficient design, load flexibility of ZEH would evolve. The operational flexibility and load shift efficiency performance of an energy efficient building, as a virtual thermal energy storage, requires further investigation. Meanwhile, the ability of a ZEH as a virtual energy storage to raise renewable self-consumption also needs to be further assessed. In particular, the availability of “behind smart meter” data enables us to examine the actual operational scenarios of the existing Japanese ZEHs; moreover, we simulate the detailed load flexible performances that manage the building as energy flexibility resources under different conditions.

The remainder of the paper is organized as follows. Section 2 provides an overview of ZEH development and studies related to operational flexibility from the distributed building sector. Section 3 introduces the examined objective, and describes the heat transfer modeling and the concept of a ZEH as virtual energy storage. Section 4 deals with the detailed operational performances of ZEHs based on the measurements and simulation model. The case study allows us to discuss the flexibility potential in detail, considering internal and external influence variables. Looking into inter-day surplus photovoltaic (PV) generation and heating load shift possibility, we analyze the impact of house preheating implementation on local PV self-consumption and room temperature profiles. Section 5 presents our conclusion.

## 3. Materials and Methods

### 3.1. Study Objective

**Figure 1:**
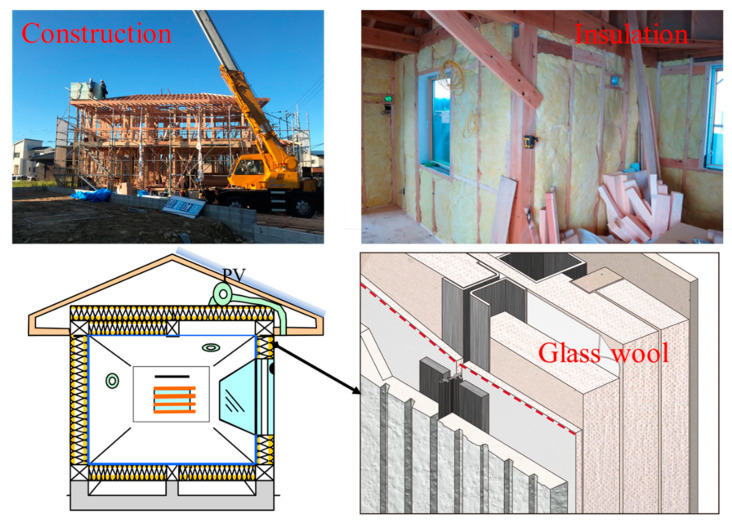
Typical scenario of the ZEH construction process with a high thermal insulation level envelope.

The location of the analyzed two-story ZEHs is in Kitakyushu City; the minimum average ambient air temperature is 2 °C in January; the maximum is 32 °C in August. The building heating period generally lasts from mid-October to mid-May of the following year. Generally, concrete and mortar are the main structural materials in conventional buildings. Figure 1 presents the typical construction scenario; the structure is composed of lightweight steel and wooden material, envelope is insulated with infilled glass wool features, with a low thermal conductivity value about 0.04 W/(m⋅K), and the envelope features high air tightness, enabling examined ZEH features with high thermal efficiency. The average overall heat loss rate of building envelope $U_{a}$ is 0.58 ${W/(m}^{2}\cdot K)$. According to the design basis, the value of the selected ZEH heat energy loss rate per floor area Q reached 1.91 ${W/(m}^{2}\cdot K)$, a conventional residential house located in the same climate region is generally built with heat loss coefficient Q 4.2 ${W/(m}^{2}\cdot K)$, $U_{a}$ is 0.87 ${W/(m}^{2}\cdot K)$; detailed information of the selected houses is described in Table 1.

**Figure 2:**
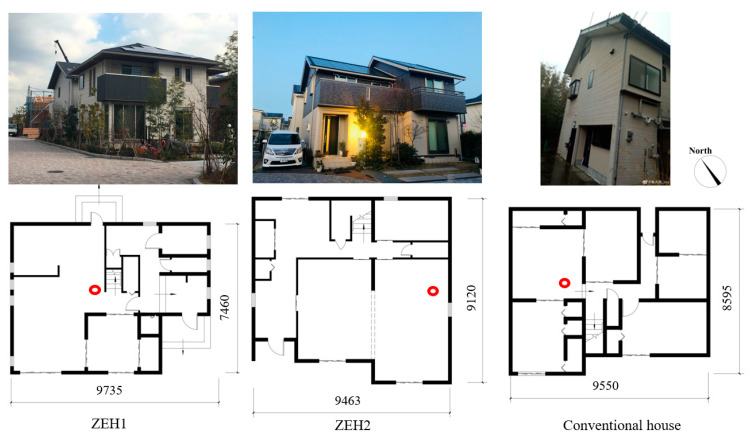
Layout of selected residential houses; the red circle presents the position of the temperature recorder.

In order to reveal the advantages of ZEHs in an internal heat energy capacity, we measured room temperature profiles of detached dwellings located in Kitakyushu City, including two ZEHs, and a conventional house built with national energy conservation standards; its Q value was 4.2, the window to wall ratio was 0.15. The first-floor layouts and positions of indoor temperature recorders (the red circle) are illustrated in Figure 2; indoor temperature profiles were recorded by TR-72wf (measurement accuracy ±0.5 °C, valid range: 0∼55 °C). The real-time electricity consumption of ZEHs were obtained from the home energy management system.

According to the definition, building energy loss is a sum of heat loss through the whole envelope, consisting of wall, rooftop, window, and floor and air ventilation loss; $U_{a}$ and Q are defined as follows:

(1) $$U_{a}=\frac{Q_{en}}{A_{en}}$$

(2) $$Q=\frac{Q_{en}+Q_{ven}}{A_{floor}}$$

where, $Q_{en}$ is the overall envelope heat energy loss, $Q_{ven}$ presents ventilation heat energy loss, $A_{en}$ is envelope area, $A_{floor}$ refers to house floor area.

According to the previous experience, the relationship between Q and $U_{a}$ of the house with 100–160 m^2^ could be described in the following linear regression mode [51]:

(3) $$Q=2.5409U_{a}+0.4345$$

### 3.2. Methodology

#### 3.2.1. House Heat Transfer Process Modeling

The dynamic thermal energy node of a house could be modeled by an equivalent thermal network made of resistances and lumped capacitances; the temperature is a voltage source, with heat flow as a current and thermal storage as a capacity. The detailed heat transfer process can be written as follows:

The heat flow from the room to the envelope, such as the wall, is written as:

(4) $$q_{rw}=\frac{T_{r}-T_{en}}{R_{r}}$$

Solar radiation exchange at external surfaces is accounted for by using the sol–air equivalent temperature, expressed as follow:

(5) $$T_{so}=T_{o}+\alpha I_{0}/h_{o}$$

where, $I_{0}$ presents solar radiation, $W/m^{2}$, ambient air temperature is $T_{o}$, °C. α is solar radiation absorptivity factor, $h_{o}$ presents coefficient of heat transfer of out surface, $W/(m^{2}\cdot K)$, $\alpha /h_{o}$ is set to 0.03 [52,53].

Similarly, the heat flow from the envelope to the outside can be written as:

(6) $$q_{en}=\frac{T_{en}-T_{so}}{R_{en}}$$

where, $R_{r}$ presents the thermal resistance between room air and exposed indoor thermal mass, $R_{en}$ is overall thermal resistance between envelope and outdoor air, considering heat transfer and ventilation, $(m^{2}\cdot K)/W$. $R_{en}$ the overall resistance of the house envelope can be estimated by designed *Q* value, $1/(Q\cdot A_{floor})-R_{r}$. $T_{en}$ is envelope temperature; solar air equivalent temperature is $T_{so}$, °C.

When the room is heated by the heat energy flow *q*, *W*, the heating equation is given as:

(7) $$q-q_{rw}=q-\frac{T_{r}-T_{en}}{R_{r}}=C_{1}\frac{dT_{air}}{dt}$$

(8) $$C_{1}=m_{air}c_{air}=\rho_{air}V_{air}c_{air}$$

where, “mair” is the indoor air mass, kg; $\rho_{air}$ is the air density, kg/m^3^; “Vair” is air volume, “cair” is the specific air heat capacity, J/(kg⋅K).

Heat transfer process through the envelope can be written as:

(9) $$q_{rw}-q_{en}=\frac{T_{r}-T_{en}}{R_{r}}-\frac{T_{en}-T_{so}}{R_{en}}=C_{2}\frac{dT_{en}}{dt}$$

(10) $$C_{2}=m_{en}c_{en}=\rho_{en}V_{en}c_{en}$$

where, “men” is the overall envelope mass, kg; $\rho_{en}$ is the density, kg/m^3^; “Ven” is volume, m^3^; “cen” is the specific heat capacity of the whole envelope, J/(kg⋅K).

Thermal behaviors of the house heat transfer can be presented in matrix form as:

(11) $$[\begin{array}{cc} C_{1} & 0\\ 0 & C_{2} \end{array}]\left[\begin{array}{c} \overset{o}{T_{r}}\\ \overset{o}{T_{en}} \end{array}\right]+\left[\begin{array}{cc} \frac{1}{R_{r}} & -\frac{1}{R_{r}}\\ -\frac{1}{R_{r}} & \frac{1}{R_{r}}+\frac{1}{R_{en}} \end{array}\right]\left[\begin{array}{c} T_{r}\\ T_{en} \end{array}\right]=\left[\begin{array}{c} q\\ \frac{T_{so}}{R_{en}} \end{array}\right]$$

#### 3.2.2. Virtual Thermal Energy Storage

**Figure 3:**
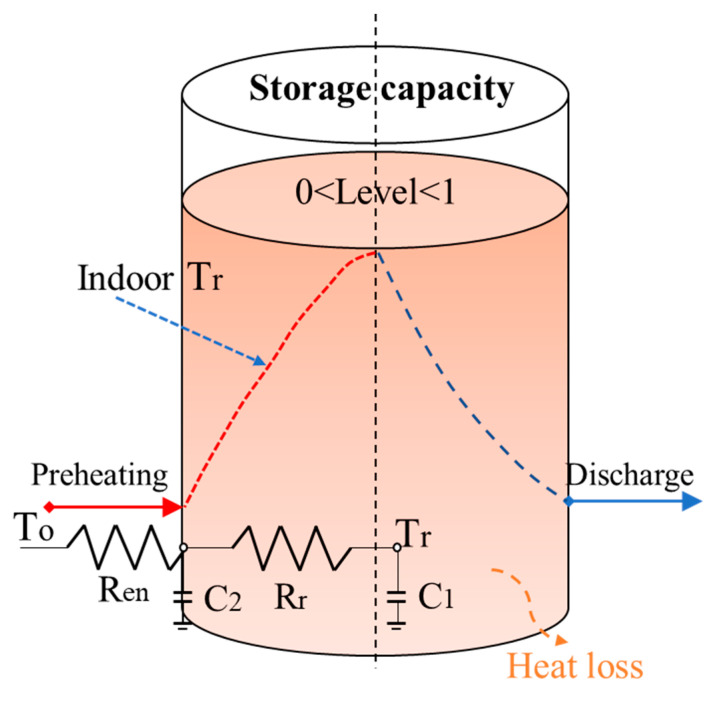
Energy node model of the virtual thermal energy storage system.

This section explains how to use building thermal mass as a virtual storage medium. Similar to the power node model of an electricity storage system, the feed-in and released heat energy flows from the building as virtual energy storage are illustrated in Figure 3. The forced charging process lasts until indoor temperature heats up to the maximum set-point temperature, as illustrated by the red dash line; the discharge process triggers when switching off the heating system. Due to the heat energy loss to the environment, the discharge process lasts until the minimum temperature value reaches within the allowed thermal comfort range; large temperature range and long duration period imply high load flexibility. The house can be partially or fully charged and its energy state of charge of the storage vessel can be normalized to the (0 1) level of the maximum heat energy storage capacity, which is determined by the possible increasing–decreasing indoor temperature range. It is important to note that cycling consumes a different amount of energy due to the indoor thermal comfort preference and room air volume. Considering the physical heat transfer process, thermal energy charging and discharging efficiencies would be influenced by an internal thermal energy loss rate during the period, which is highly associates with the weather condition, building physical thermal properties, and set-point operative temperature.

## 4. Results and Discussions

### 4.1. Measured Operational Performances

**Figure 4:**
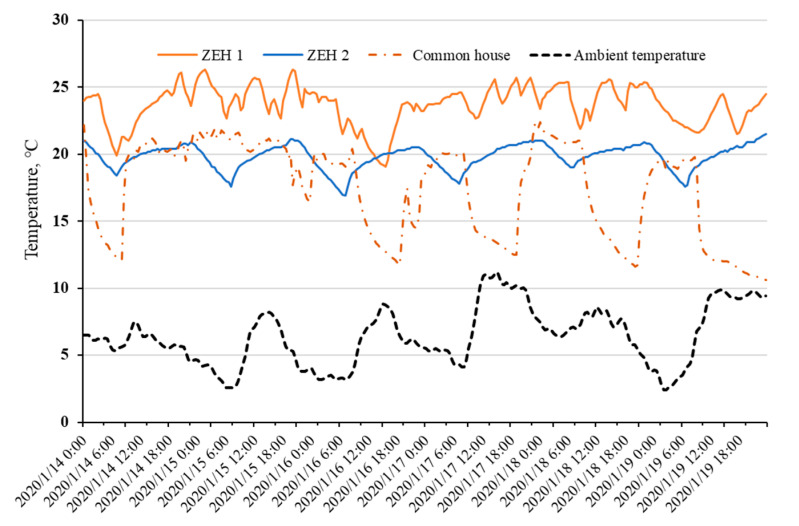
Comparison of measured indoor air temperature profiles among ZEHs, common house from 14 January to 19 January 2020.

Detailed comparisons of measured indoor temperature profiles are described in Figure 4; there is less of an indoor air temperature drop rate after switching off the air conditioner in ZEHs. A larger range of indoor temperature fluctuations of the conventional detached house is depicted in the dash dot line; well-insulated ZEHs show the ability to retain indoor heat energy. In contrast to the conventional house, ZEHs obviously prolonged the delay time of the heating system within the comfort temperature range.

**Figure 5:**
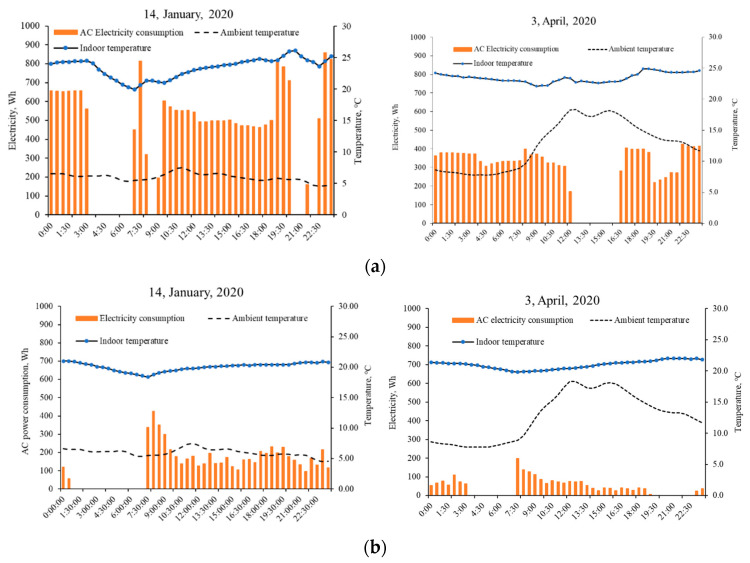
Variations of measured daily room air temperature, ambient air temperature, and corresponding air conditioner electricity consumption profiles of ZEH 1 (**a**) and ZEH 2 (**b**).

The measured real-time electricity usage of a room air conditioner can help provide better understanding of the dynamic behaviors of the energy performances of ZEHs, induced by the occupied schedule and ambient temperature. As displayed in Figure 5, the electricity consumption of ZEHs for room heating has a close relationship with set-point operative temperature; indoor thermal comfort preferences of residents would influence the heating power consumption. It resulted in a peak electricity load (30-min interval) to lift the room temperature. The lower outdoor temperature increased electricity consumption due to the increased heating load and higher energy loss. ZEHs featured great ability to retain indoor heat energy; indoor air temperature of ZEHs could be kept within the comfort zone, with less heating power consumption on a warm day (in this case, 3 April 2020), even after switching off the air conditioner.

**Figure 6:**
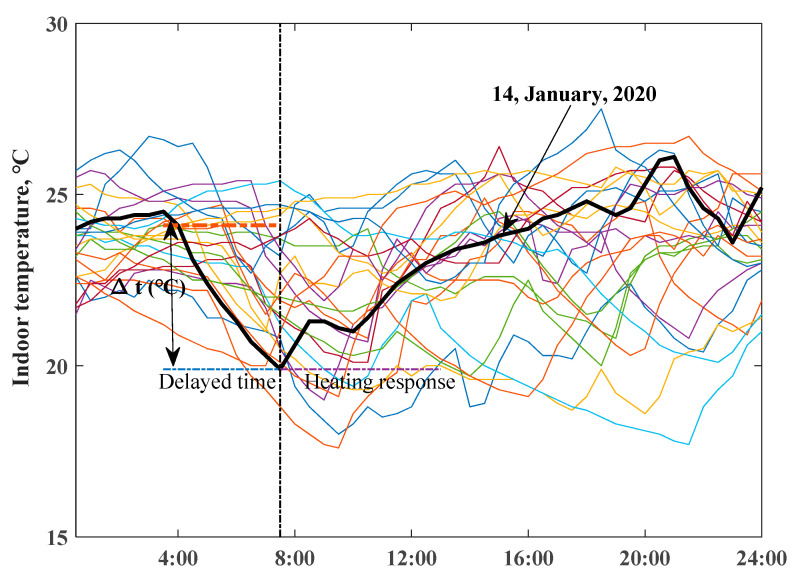
Daily indoor air temperature profiles of ZEH 1, bold black line presents the selected typical day.

**Figure 7:**
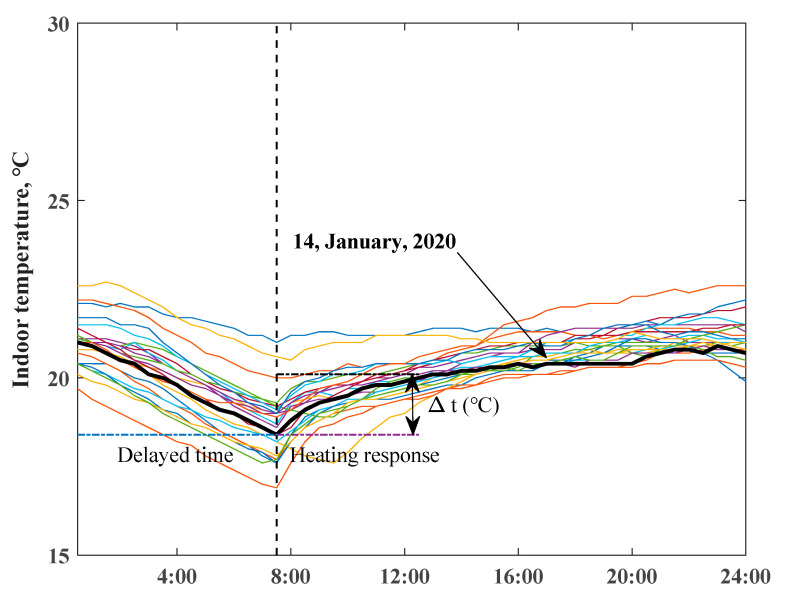
Daily indoor air temperature profiles of ZEH 2, bold black line presents the selected typical day.

The chosen scenarios for measured daily indoor temperature profiles of two ZEHs from 1 January to 24 January, 2020 are depicted in Figure 6 and Figure 7. The variations in room temperatures of ZEH are clearly illustrated—they were highly determined by the occupied behaviors of residents. The daily lowest temperature of ZEH 2 mainly occurred in the early morning period. The typical heating up and delayed behaviors of the room temperature are revealed in bold black lines.

### 4.2. Simulation Results

More case studies are needed to analyze the detailed impacts of internal and external factors on indoor temperature and load flexibility performance. We built the simulation model based on the detailed structure physical parameters of the ZEH 1, facing south, the first-floor area was 66.4 m^2^, the height was 3.2 m. The following simulations were carried out based on input parameter values, as depicted in Table 2.

**Figure 8:**
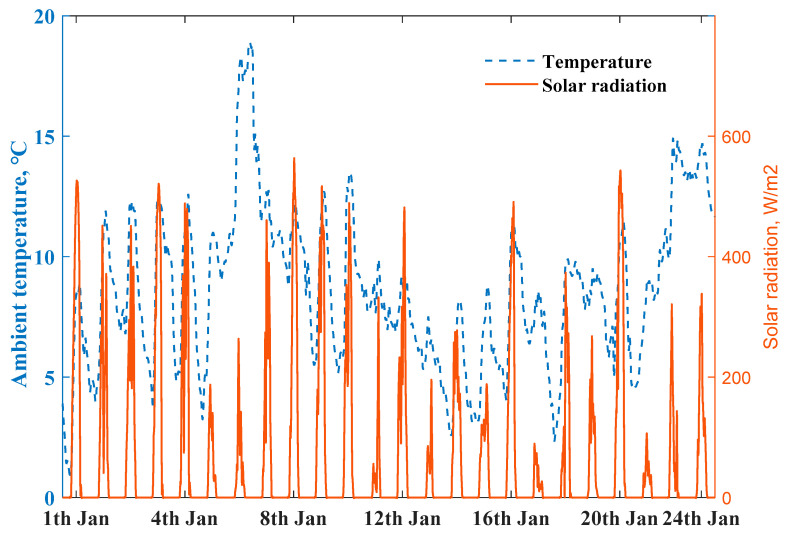
Solar radiation and ambient air temperature profiles.

**Figure 9:**
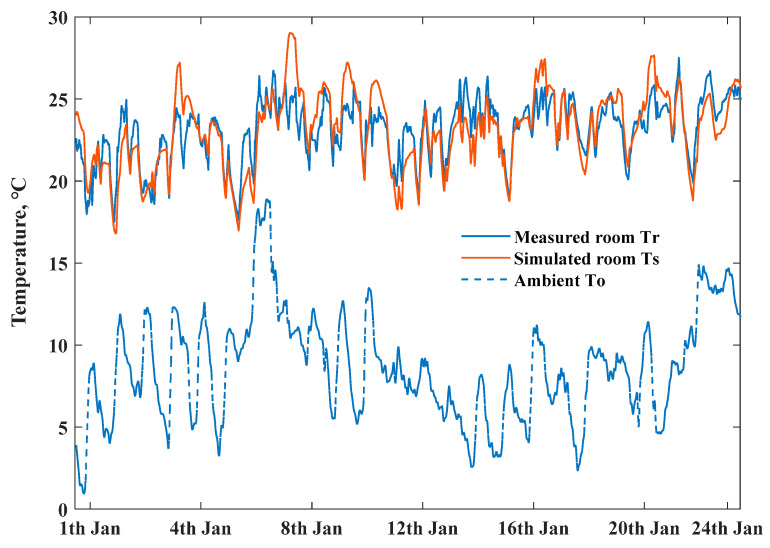
Measured and simulated room air temperature profiles of ZEHs with heat loss coefficient, Q is 1.91 $W/(m^{2}\cdot K)$.

The heat generation of the air conditioner and room appliances electricity consumption are summed as indoor heat energy gain.$R_{en}$ is difficult to calculate accurately considering the complex material and components of the house envelope, we used Q value of the house’s whole envelope to estimate its thermal resistance, to simulate the heat transfer above the RC model. The outside weather conditions are presented in Figure 8; air conditioner heat generation and measured indoor appliances electricity consumption are the boundary input. The working performance of the air conditioner has a close relationship with ambient temperature, the coefficient performance of the air conditioner is determined by minimizing the root mean square error between the measured and simulated indoor temperature of the ZEH. We used the above thermal response model to simulate the scenario of the indoor temperature built in a MATLAB environment. Figure 9 depicts the measured and simulated indoor temperatures profiles at 1-min intervals with the same heating power input from 1 to 24 January 2020; the simulated room temperature profile (black line) with 1.91 $W/(m^{2}\cdot K)$ heat loss coefficient (designed value) could fit well with an actual measured living room temperature of ZEH 1.

**Figure 10:**
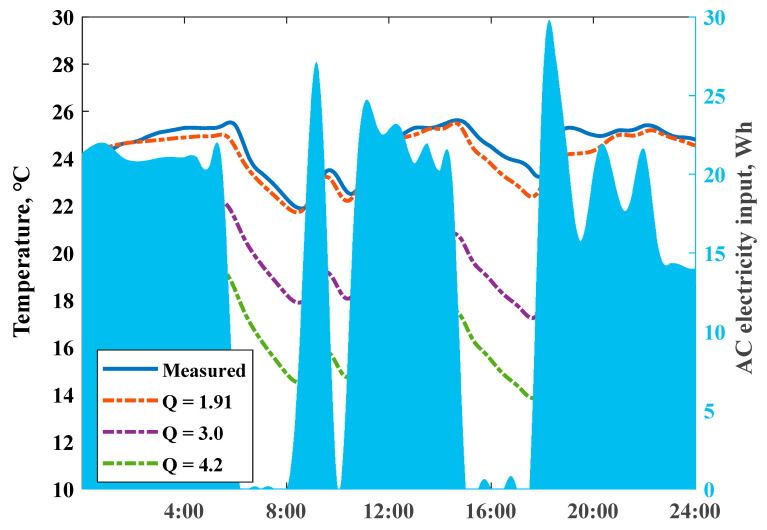
Scenario of measured and simulated indoor air temperatures with unchanged AC heating power input under different Q values.

As shown in Figure 10, actual measured room temperature and dynamic thermal process of the house on a single day (18, January,2020) with intermittent heating supply was simulated with different heat loss coefficient values. The right hand y-axis presents the air conditioner electricity consumption; detailed impacts (after switching off the air conditioner at room temperature) were compared. The 3.0 and 4.2 are typical Q values in residential energy-saving design standards (1999 and 2013) in this region. Results indicated that houses with lower Q values had an advantage in minimizing daily room temperature fluctuations, attributed to the ability to store inside heat for a longer duration.

**Figure 11:**
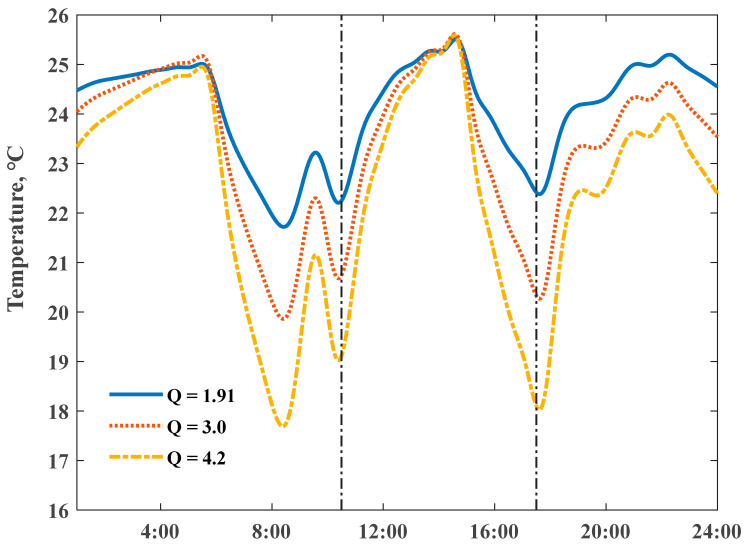
Impact of heat loss coefficient Q values on simulated indoor air temperature profiles, on 18 January 2020.

We fixed the maximum set-point indoor temperature to 25.5 °C, and then simulated the daily temperature profiles by scaling the electricity consumption of the air conditioners with different structural thermal insulation levels; corresponding power input scaling ratios reached 1.0, 1.66, and 2.30, respectively. The stronger ability of a ZEH in storing internal heat could smooth net load variations. Figure 11 reveals the detailed comparisons of indoor temperature profiles; the descent slopes responding to switch off the heating power highly depended on the Q values. The domain between the dash dot lines could present whole preheat charging and a delayed discharging cycle; a virtual energy storage tank–well-insulated house presented a large potential to shift stored heat energy over a longer time, and “damped” the variations of the daily room temperatures under intermittent heating supply conditions. A ZEH with the Q value 1.91 $W/(m^{2}\cdot K)$ allowed for a shorter room heat-up response time and extended the duration period of the indoor comfort temperature range.

### 4.3. Operational Load Flexibility

**Figure 12:**
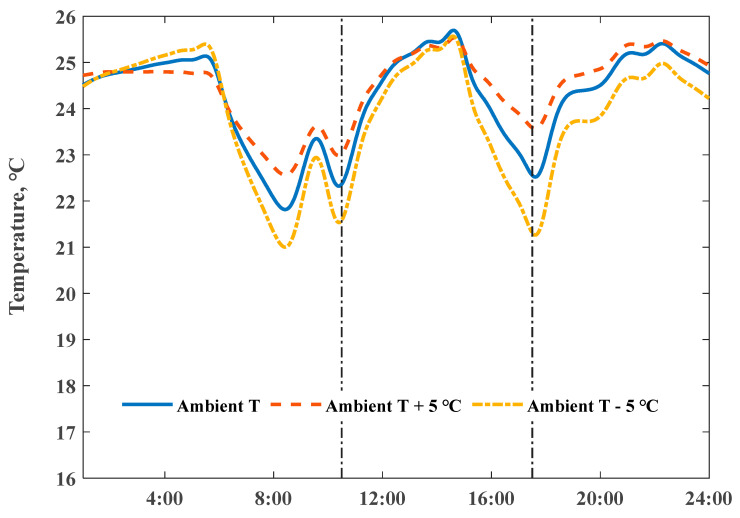
Impact of ambient temperature on the simulated indoor air temperature profiles with a Q value of 1.91 $W/(m^{2}\cdot K)$.

Not all of the stored heat energy inside of the envelope could be used to maintain the room temperature during the delayed period—part of the stored heat energy was lost to the outside environment during the heat release process, which has a close relationship with the amount of stored heat energy. To reveal this effect, we rescaled the heating power input to maintain the fixed maximum indoor temperature, and simulated the variations of indoor temperatures induced by changing the ambient temperature; the actual daily average ambient temperature was 6.9 °C (solid line in Figure 12). As observed in Figure 12, results show that the indoor temperature descent rate, after switching off the heating system, presented an obvious negative relationship with outdoor–indoor temperature difference.

**Figure 13:**
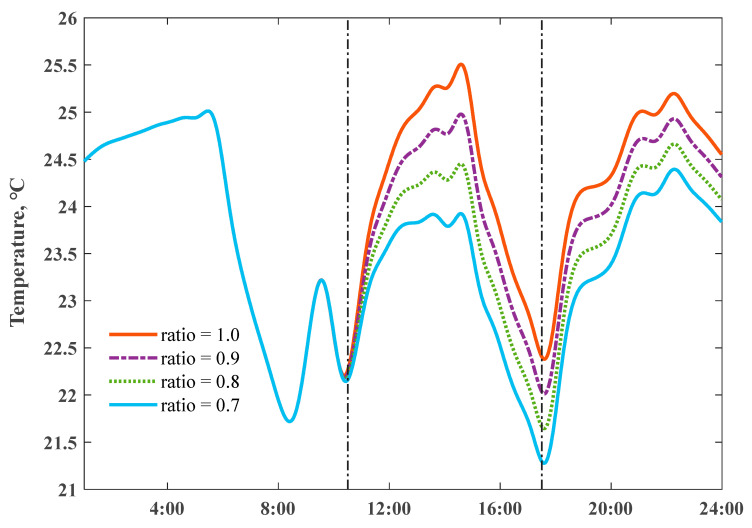
Changes in indoor air temperature profiles under different heating power inputs on 18 January 2020.

Meanwhile, we used the above RC model to simulate the scenario of indoor temperature profiles through various heating power input ratios, from 9:30 to 18:00 on 18 January 2020, as shown in Figure 13. It allowed investigating the influence of the power input in operative temperature; the indoor temperature delayed state showed that the heat loss rate was large at the initial switch-off stage of the heating system, especially at a higher room temperature. We can confirm that the building’s virtual energy storage tank’s high set point, with fully charged temperature, could decrease thermal storage efficiency.

### 4.4. Load Shift Potential

**Figure 14:**
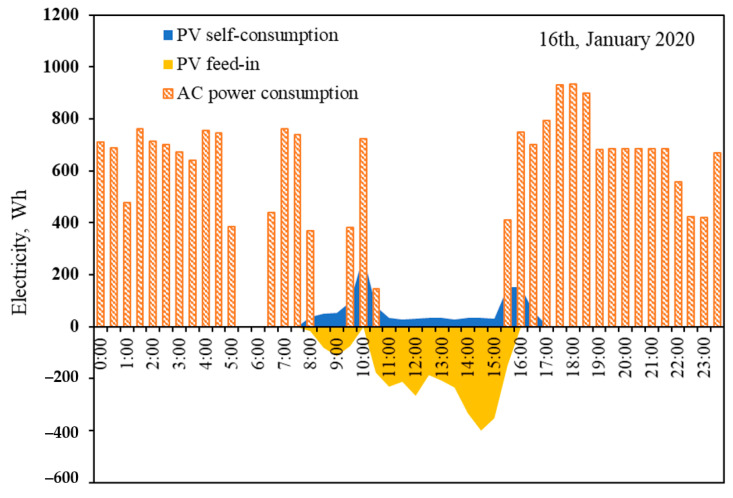
Typical scenario of air conditioner load, PV local consumption, and feed-in power.

PV generation is the most available type of renewable energy in a ZEH; the measured power flows of ZEH 1 with nominal PV capacity 4.8 kWp, including air conditioner (AC) power consumption, self PV consumption, and grid feed-in power at the 30-min interval, on 16 January 2020, are illustrated in Figure 14. The mismatch between simultaneous demand and PV production profiles led a large ratio of PV generation, and had to be fed into the grid from 13:00 to 15:00 There is a (continuous) decreasing trend of PV feed-in tariff in Japan; if a PV system with less than 10 kW capacity reached 21 Yen/kWh in 2020, the price would drop to 7 Yen/kWh after the contract period of 10 years [54]. Considering the current daytime electricity market price is about 24 Yen/kWh, a cost-saving potential was available by raising the local PV self-consumption ratio. A ZEH with a large ability to retain inside heat creates the chance to increase the penetration of local PV generation by active heating load shift.

**Figure 15:**
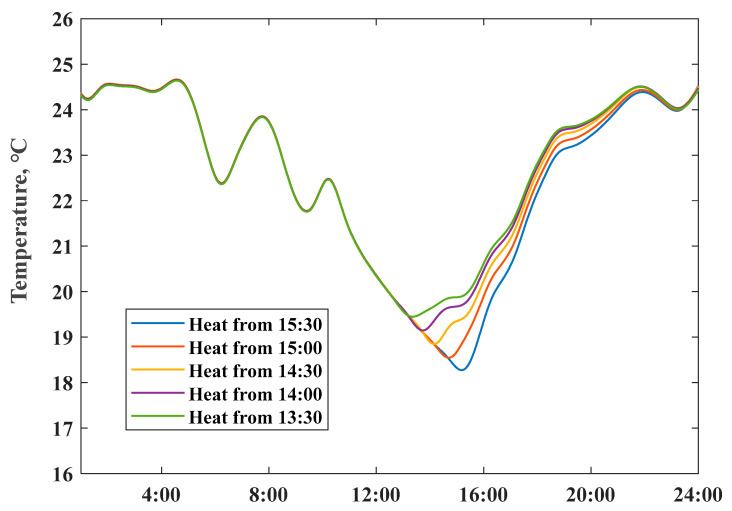
Simulated indoor air temperature profile by adjusting preheating beginning time.

To explore the potential of raising the on-site PV consumption ratio by preheating the house, we simulated the room temperature by adjusting the preheating start time during an unoccupied period, meanwhile, scaled the power input from 15:30 to 24:00 to limit the maximum room temperature within the comfort region. Figure 15 shows the simulated room temperature profiles of different preheating events; the real-time surplus PV production was utilized as a preheating power source. Details on the surplus PV production consumption as the preheating power input and reduction of the grid import electricity from 15:30 to 24:00 are summarized in Table 3. Results indicate that the preheating approach could shift at approximately 50% of total surplus PV generation input, to replace later electricity consumption from 15:30 to 24:00, verified the effectiveness of heating load shifting in raising local PV self-consumption ratio.

**Figure 16:**
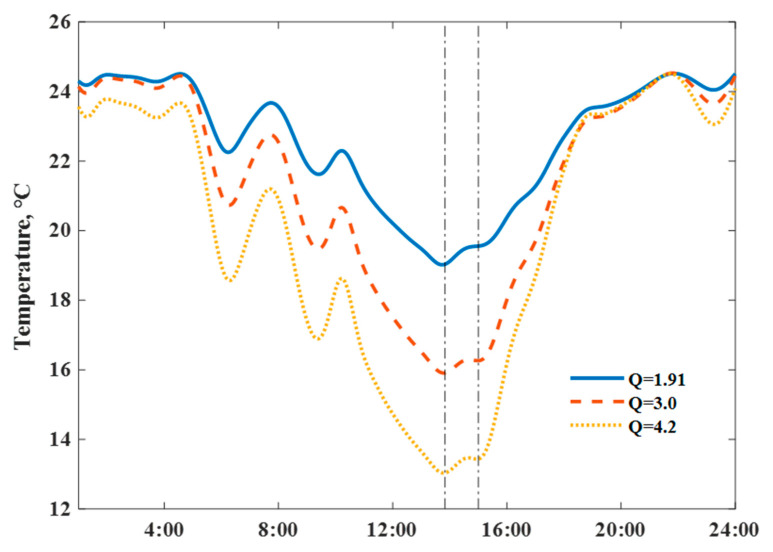
Simulated preheat response behaviors of house with different heat loss coefficient values.

We fixed the daily maximum indoor temperature and rescaled the heating power input under unchanged air conditioning occupied behaviors. Figure 16 presents the simulated scenarios of room temperatures with different heat loss coefficient values Q after preheat is implemented (starting from 14:00). Results show that preheating might help maintain whole day comfort temperature at a ZEH, with a low heat loss coefficient value. We could observe the effects of room preheating on room temperature lift (temperature difference between the solid and dashed lines), obvious under a low Q value, despite the relatively high operative temperatures. The scenario highlighted the heat energy storage ability of a well-insulated ZEH model, reducing the energy consumption.

## 5. Conclusions

With continuous improvements of buildings’ energy efficient standards, this research investigated the operational performances of Japanese ZEHs, based on on-site measurements. By modeling the house as a virtual energy storage medium—detailed operational energy flexibility performances of ZEHs were analyzed under different scenarios. The main findings are summarized as follows:

The interactions between envelope energy efficiency and indoor temperature variations are observed in heating periods. Compared with the selected conventional house, both measured and simulated results confirmed that ZEHs with lower heat loss coefficient values presented clear advantages in “damping” the variations of indoor air temperatures. We clearly observed that ZEHs shorten the indoor heating-up response times and prolong the delayed period within an acceptable thermal comfort range. Moreover, ZEHs show great ability in reducing the heat loss rate through the whole envelope, under high indoor air temperature.

Simulation results of the developed house thermal RC model fit well with actual measurements. Results revealed the influences of thermal insulation levels, ambient conditions, and set-point indoor temperatures in regards to a house’s heat retention ability. Energy flexibility of a ZEH’s structural thermal mass, acting as a virtual thermal storage medium, was investigated by heating-up the response and delayed duration characteristics of indoor air temperatures. Results from case studies indicated that high indoor set-point temperatures could lead to a rapid temperature drop rate after switching off the heating system; thus, decreasing the virtual thermal storage efficiency. A (more than) 20% reduction of heating power consumption was achieved when a 2 °C adjustable range of indoor set-point temperature occurred.

In light of the low-energy loss rate and dropping PV feed-in tariff value, a ZEH with high thermal insulation levels created the opportunity to consume excess PV generation by a real-time load shift. Based on the developed simulation model, results verified the effectiveness of the coupling room preheating approach and surplus PV generation consumption during an unoccupied period. Approximate 50% of consumed PV generation could replace the power consumption of room heating during occupancy time. Implementing a house preheating strategy raised the local PV self-consumption ratio and increased the overall energy consumption.

In future work, more data will be collected to assess the cooling flexible performance of a ZEH in the summer period. A single house has limited load shift potential. After acquiring better understanding of the effects of the various physical and environmental factors, we would conduct an integrated flexible building group as a virtual thermal energy storage medium, to facilitate high penetration levels of variable renewables in a multi-energy system district.

## Figures and Tables

**Table 1:** Main characteristics of the selected ZEHs.

Established Year	2017
Structural material	Lightweight steel and wooden skeleton, infill structure, 2 stories
Envelope heat loss coefficient	Q: 1.91 W/(m^2^ K), $U_{a}$: 0.58 W/(m^2^ K)
Thermal insulation material and characteristic	Wall: glass wool 120 mm and glass board 12 mm; roof: glass wall 100 mm; floor: glass wall 67 mm; thermal conductivity 0.04 W/(m K)
Ventilation rate	Mechanical ventilation, 0.5 ac/h
Window	Low-E pair glass with plastic combined aluminum sash
Window-to-wall ratio	ZEH 1: 0.16, ZEH 2: 0.18

**Table 2:** Calculated RC model parameters.

Variables	Room Air Volume, m^3^	Structure Volume, m^3^	C1, kJ/K	C2, kJ/K
Value	210	8	2540	5000

**Table 3:** Simulation results of house preheat cases.

Variables	From 15:00	From 14:30	From 14:00	From 13:30
Preheat power input (PV generation), Wh	498	897	1230	1463
Amount of replaced heating power, Wh	201	433	665	839

## Data Availability

Not applicable.

## Nomenclature

Aen	Total envelope area, m^2^
Afloor	House floor area, m^2^
C1	Room air heat capacity, kJ/K
C2	Envelope heat capacity, kJ/K
Ua	Thermal transmittance of the envelope, W/(m^2^*K)
Q	Building heat loss coefficient, W/(m^2^*K)
Qen	Overall envelope heat energy loss per degree, W/K
Qven	Ventilation heat energy loss per degree, W/K
Io	Solar radiation, W/m^2^
qrw	Heat flow from the room to the envelope, W/m^2^
qen	Heat flow from the envelope to the outside, W/m^2^
q	Indoor heat energy gain, W/m^2^
Rr	Thermal resistance between room air and exposed indoor thermal mass, (m^2^*K)/W
Ren	Overall thermal resistance between envelope and outdoor air and ventilation, (m^2^*K)/W
ho	Coefficient of heat transfer of out surface, W/(m^2^*K)
To	Ambient temperature, °C
Ten	Envelope temperature, °C
Tr	Room air temperature, °C
Tso	Solar air equivalent temperature, °C
Vair	Room air volume, m^3^
Ven	Room envelope volume, m^3^
cair	The specific air heat capacity, J(kg*K)
cen	The specific envelope heat capacity, J(kg*K)
mair	Indoor air mass, kg
men	Envelope mass, kg
α	Solar radiation absorptivity factor
ρair	Air density, kg/m^3^
ρen	Overall envelope density, kg/m^3^
ZEH	Zero energy house
PV	Photovoltaic

## References

[1] Oki R., Tsuneoka Y., Yamaguchi S., Sugano S., Watanabe N., Akimoto T., Hayashi Y., Wakao S., Tanabe S.-I. (2019). Renovating a house to aim for net-zero energy, thermal comfort, energy self-consumption and behavioural adaptation: A method proposed for enemane house 2017. Energy Build..

[2] Li Y., Gao W., Zhang X., Ruan Y., Ushifusa Y., Hiroatsu F. (2020). Techno-economic performance analysis of zero energy house applications with home energy management system in Japan. Energy Build..

[3] Stinner S., Huchtemann K., Müller D. (2016). Quantifying the operational flexibility of building energy systems with thermal energy storages. Appl. Energy.

[4] Wang H., Wang S., Tang R. (2019). Development of grid-responsive buildings: Opportunities, challenges, capabilities and applications of HVAC systems in non-residential buildings in providing ancillary services by fast demand responses to smart grids. Appl. Energy.

[5] Li Y., Gao W., Ruan Y. (2018). Performance investigation of grid-connected residential PV-battery system focusing on enhancing self-consumption and peak shaving in Kyushu, Japan. Renew. Energy.

[6] Le Dréau J., Heiselberg P. (2016). Energy flexibility of residential buildings using short term heat storage in the thermal mass. Energy.

[7] Klein K., Herkel S., Henning H.-M., Felsmann C. (2017). Load shifting using the heating and cooling system of an office building: Quantitative potential evaluation for different flexibility and storage options. Appl. Energy.

[8] Tulabing R., Yin R., DeForest N., Li Y., Wang K., Yong T., Stadler M. (2016). Modeling study on flexible load’s demand response potentials for providing ancillary services at the substation level. Electr. Power Syst. Res..

[9] Sweetnam T., Fell M., Oikonomou E., Oreszczyn T. (2018). Domestic demand-side response with heat pumps: Controls and tariffs. Build. Res. Inf..

[10] Taibi E., Nikolakakis T., Gutierrez L., Fernandez C., Kiviluoma J., Rissanen S., Lindroos T.J. (2018). Power System Flexibility for the Energy Transition: Part 1, Overview for Policy Makers.

[11] Li P.-H., Pye S. (2018). Assessing the benefits of demand-side flexibility in residential and transport sectors from an integrated energy systems perspective. Appl. Energy.

[12] Olivella-Rosell P., Lloret-Gallego P., Munné-Collado Í., Villafafila-Robles R., Sumper A., Ottessen S., Rajasekharan J., Bremdal B. (2018). Local flexibility market design for aggregators providing multiple flexibility services at distribution network level. Energies.

[13] Ansarin M., Ghiassi-Farrokhfal Y., Ketter W., Collins J. (2020). The economic consequences of electricity tariff design in a renewable energy era. Appl. Energy.

[14] Lin Y., Zhong S., Yang W., Hao X., Li C.-Q. (2020). Towards zero-energy buildings in China: A systematic literature review. J. Clean. Prod..

[15] Esbensen T.V., Korsgaard V. (1977). Dimensioning of the solar heating system in the zero energy house in Denmark. Sol. Energy.

[16] Yu Z., Gou Z., Qian F., Fu J., Tao Y. (2019). Towards an optimized zero energy solar house: A critical analysis of passive and active design strategies used in Solar Decathlon Europe in Madrid. J. Clean. Prod..

[17] Kosonen A., Keskisaari A. (2020). Zero-energy log house—Future concept for an energy efficient building in the Nordic conditions. Energy Build..

[18] Vieira F.M., Moura P.S., de Almeida A.T. (2017). Energy storage system for self-consumption of photovoltaic energy in residential zero energy buildings. Renew. Energy.

[19] Wang R., Feng W., Wang L., Lu S. (2021). A comprehensive evaluation of zero energy buildings in cold regions: Actual performance and key technologies of cases from China, the US, and the European Union. Energy.

[20] Schill W.-P., Zerrahn A. (2020). Flexible electricity use for heating in markets with renewable energy. Appl. Energy.

[21] Li Y., Zhang X., Gao W., Ruan Y. (2020). Capacity credit and market value analysis of photovoltaic integration considering grid flexibility requirements. Renew. Energy.

[22] Vigna I., Pernetti R., Pasut W., Lollini R. (2018). New domain for promoting energy efficiency: Energy flexible building cluster. Sustain. Cities Soc..

[23] Jensen S.Ø., Marszal-Pomianowska A., Lollini R., Pasut W., Knotzer A., Engelmann P., Stafford A., Reynders G. (2017). IEA EBC annex 67 energy flexible buildings. Energy Build..

[24] Bechtel S., Rafii-Tabrizi S., Scholzen F., Hadji-Minaglou J.-R., Maas S. (2020). Influence of thermal energy storage and heat pump parametrization for demand-side-management in a nearly-zero-energy-building using model predictive control. Energy Build..

[25] Fitzpatrick P., D’Ettorre F., De Rosa M., Yadack M., Eicker U., Finn D.P. (2020). Influence of electricity prices on energy flexibility of integrated hybrid heat pump and thermal storage systems in a residential building. Energy Build..

[26] Li Y., Gao W., Ruan Y., Ushifusa Y. (2018). Demand response of customers in Kitakyushu smart community project to critical peak pricing of electricity. Energy Build..

[27] Shen L., Li Z., Sun Y. (2016). Performance evaluation of conventional demand response at building-group-level under different electricity pricings. Energy Build..

[28] Yu Z., Lu F., Zou Y., Yang X. (2020). Quantifying the flexibility of lighting systems by optimal control in commercial buildings: Insight from a case study. Energy Build..

[29] Barone G., Buonomano A., Forzano C., Giuzio G.F., Palombo A. (2020). Increasing self-consumption of renewable energy through the building to vehicle to building approach applied to multiple users connected in a virtual micro-grid. Renew. Energy.

[30] Georges E., Cornélusse B., Ernst D., Lemort V., Mathieu S. (2017). Residential heat pump as flexible load for direct control service with parametrized duration and rebound effect. Appl. Energy.

[31] Williams S., Short M., Crosbie T. (2018). On the use of thermal inertia in building stock to leverage decentralised demand side frequency regulation services. Appl. Therm. Eng..

[32] Asadinejad A., Rahimpour A., Tomsovic K., Qi H., Chen C.-F. (2018). Evaluation of residential customer elasticity for incentive based demand response programs. Electr. Power Syst. Res..

[33] Violante W., Canizares C.A., Trovato M.A., Forte G. (2020). An energy management system for isolated microgrids with thermal energy resources. IEEE Trans. Smart Grid.

[34] Chassin D.P., Stoustrup J., Agathoklis P., Djilali N. (2015). A new thermostat for real-time price demand response: Cost, comfort and energy impacts of discrete-time control without deadband. Appl. Energy.

[35] Short M., Rodriguez S., Charlesworth R., Crosbie T., Dawood N. (2019). Optimal dispatch of aggregated HVAC units for demand response: An industry 4.0 approach. Energies.

[36] Danza L., Belussi L., Meroni I., Salamone F., Floreani F., Piccinini A., Dabusti A. (2016). A simplified thermal model to control the energy fluxes and to improve the performance of buildings. Energy Procedia.

[37] Ghiaus C., Ahmad N. (2020). Thermal circuits assembling and state-space extraction for modelling heat transfer in buildings. Energy.

[38] Hurwitz Z.L., Dubief Y., Almassalkhi M. (2020). Economic efficiency and carbon emissions in multi-energy systems with flexible buildings. Int. J. Electr. Power Energy Syst..

[39] Weiß T., Fulterer A.M., Knotzer A. (2018). Energy flexibility of domestic thermal loads—A building typology approach of the residential building stock in Austria. Adv. Build. Energy Res..

[40] Jin X., Mu Y., Jia H., Wu J., Jiang T., Yu X. (2017). Dynamic economic dispatch of a hybrid energy microgrid considering building based virtual energy storage system. Appl. Energy.

[41] Masy G., Georges E., Verhelst C., Lemort V., André P. (2015). Smart grid energy flexible buildings through the use of heat pumps and building thermal mass as energy storage in the Belgian context. Sci. Technol. Built Environ..

[42] Oliveira Panão M.J.N., Mateus N.M., Carrilho da Graça G. (2019). Measured and modeled performance of internal mass as a thermal energy battery for energy flexible residential buildings. Appl. Energy.

[43] Reynders G., Amaral Lopes R., Marszal-Pomianowska A., Aelenei D., Martins J., Saelens D. (2018). Energy flexible buildings: An evaluation of definitions and quantification methodologies applied to thermal storage. Energy Build..

[44] Kamel E., Iuldasheva A. The Effectiveness of HVAC Demand Response Control on Buildings with Low and High Thermal Insulation. Proceedings of the 2019 Buildings XIV International Conference.

[45] Balint A., Kazmi H. (2019). Determinants of energy flexibility in residential hot water systems. Energy Build..

[46] Ulbig A., Andersson G. (2015). Analyzing operational flexibility of electric power systems. Int. J. Electr. Power Energy Syst..

[47] Kathirgamanathan A., Péan T., Zhang K., De Rosa M., Salom J., Kummert M., Finn D.P. (2020). Towards standardising market-independent indicators for quantifying energy flexibility in buildings. Energy Build..

[48] Harder N., Qussous R., Weidlich A. (2020). The cost of providing operational flexibility from distributed energy resources. Appl. Energy.

[49] De Coninck R., Helsen L. (2016). Quantification of flexibility in buildings by cost curves—Methodology and application. Appl. Energy.

[50] Junker R.G., Azar A.G., Lopes R.A., Lindberg K.B., Reynders G., Relan R., Madsen H. (2018). Characterizing the energy flexibility of buildings and districts. Appl. Energy.

[51] Institute H.P.D. (2018). Investigation of the Relationship between House Q and UA Value.

[52] Al-Saud K.A. (2009). Measured versus calculated roof peak sol-air temperature in hot-arid regions. Red.

[53] Tuck N.W., Zaki S.A., Hagishima A., Rijal H.B., Yakub F. (2020). Affordable retrofitting methods to achieve thermal comfort for a terrace house in Malaysia with a hot–humid climate. Energy Build..

[54] Ministry of Economy, Trade and Industry Feed in Tariff for Renewable Energy in Japan. https://www.meti.go.jp/shingikai/santeii/pdf/063_01_00.pdf.

